# First whole genome based microsatellite DNA marker database of tomato for mapping and variety identification

**DOI:** 10.1186/1471-2229-13-197

**Published:** 2013-12-04

**Authors:** Mir A Iquebal, Sarika, Vasu Arora, Nidhi Verma, Anil Rai, Dinesh Kumar

**Affiliations:** 1 Centre for Agricultural Bioinformatics, Indian Agricultural Statistics Research Institute, Library Avenue, New Delhi 110012, India; 2 Germplasm Exchange Unit, National Bureau of Plant Genetic Resources (NBPGR), New Delhi 110012, India

**Keywords:** Markers, Microsatellite, MISA, Primer, Tomato, Variety identification

## Abstract

**Background:**

The cultivated tomato is second most consumed vegetable of the world and is an important part of a diverse and balanced diet as a rich source of vitamins, minerals, phenolic antioxidants and antioxidant lycopene having anti-cancer properties. To reap benefit of genomics of the domestic tomato (*Solanum lycopersicum* L.) unravelled by Tomato Genome Consortium (The Tomato Genome Consortium, 2012), the bulk mining of its markers in totality is imperative and critically required. The solgenomics has limited number of microsatellite DNA markers (2867) pertaining to solanaceae family. As these markers are of linkage map having relative distance, the choice of selected markers based on absolute distance as of physical map is missing. Only limited microsatellite markers with limitations are reported for variety identification thus there is a need for more markers supplementing DUS test and also for traceability of product in global market.

**Description:**

We present here the first whole genome based microsatellite DNA marker database of tomato, *TomSatDB* (Tomato MicroSatellite Database) with more than 1.4 million markers mined *in-silico*, using *MIcroSAtellite* (MISA) tool. To cater the customized needs of wet lab, features with a novelty of an automated primer designing tool is added. *TomSatDB* (http://cabindb.iasri.res.in/tomsatdb), a user-friendly and freely accessible tool offers chromosome wise as well as location wise search of primers. It is an online relational database based on “three-tier architecture” that catalogues information of microsatellites in MySQL and user-friendly interface developed using PHP (Hypertext Pre Processor).

**Conclusion:**

Besides abiotic stress, tomato is known to have biotic stress due to its susceptibility over 200 diseases caused by pathogenic fungi, bacteria, viruses and nematodes. These markers are expected to pave the way of germplasm management over abiotic and biotic stress as well as improvement through molecular breeding, leading to increased tomato productivity in India as well as other parts of the world. In era of IPR the new variety can be identified based on allelic variation among varieties supplementing DUS test and product traceability.

## Background

Tomato (*Solanum lycopersicon* L.), a new world solanaceous plant is an excellent model for plant genomic research. The genus *Solanum* is one of the largest angiosperm genera and the genome has 35,000 genes spread over 12 chromosomes and has few high copy number long terminal repeat (LTR) retrotransposons and largely comprised of low-copy DNA [1]. The genome of tomato has been sequenced by The Tomato Genome Consortium in 2012 [1]. A draft sequence of its closest wild relative, i.e. *Solanum pimpinellifolium* and the potato genome (*Solanum tuberosum* L.) has also been reported depicting the extent and pattern of similarities and dissimilarities among the three genomes. The tomato genome sequence will have implications on other plant species viz., strawberries, melons, apple etc., which share some characteristics with tomato. Especially common information related to gene and pathway involved in fruit ripening can be potentially applied to other crops also leading to improved fruit quality and reduced cold chain management costs [2].

The leading tomato producing countries of the world are China, USA, India, Turkey, Egypt, and Italy [3]. Globally, USA (23.2%), Germany (16.5%), Russian Federation (9.6%) and United Kingdom (8.3%) are top countries importing tomatoes while Netherlands is the biggest exporter of tomatoes, exporting over 910 346 tons a year and accounting for 20.6% of world export market in tomatoes [4].

Worldwide, tomatoes are an important part of a diverse and balanced diet as a rich source of vitamins, minerals, phenolic antioxidants and anti-oxidant lycopene having anti-cancer properties [5,6]. A major constraint in tomato production is the loss incurred due to several diseases. Tomato is known to be susceptible to over 200 diseases caused by pathogenic fungi, bacteria, viruses and nematodes [7]. To accelerate conventional plant breeding, Marker Assisted Selection (MAS) with more than 80 resistance genes to major classes of pathogens (fungal, bacterial, virus and nematode) have been used extensively for pyramiding resistance genes [8,9]. The other essential characteristics for tomato improvement are development of cultivars with broad adaptability, earliness and fruit quality. Over 75,000 accessions of the cultivated and wild species of tomato are maintained in Genebank around the world [10] but relative and absolute differentiation of these accessions need microsatellite (STR) markers. Thus, these bulk whole genome based STR markers are needed for mapping, variety identification and product traceability.

Earlier *in silico* works for STR mining were not based on whole genome and thus invariably yielded low/very less number of markers, for example just 80 STR were found in entire gene bank search over 2000 sequences. Though the markers were minimum but are highly potential in distinguishing closely related cultivars of tomato [11]. Reported marker density based on *in vivo* method is relatively less on every chromosome, for example 12^th^ chromosome has just 37 [12]. For plant variety identification along with degree of admixture, STR is always preferred if they are in multiplex mode (for example, Basmati and non-Basmati rice can be differentiated by 8 plex/ single cocktail based PCR) [13]. Such multiplex designing needs much more number of markers to design multiplex with thermodynamic compatibility, which can be accomplished from our large marker dataset. SGN database is having various classes of markers including STR which are chromosome wise and distance wise based on LOD (Logarithm of the Odds) score [14]. Uniformly distributed markers over genome with an average spacing of 10.0 cM [15] and 1.2 cM (ca. 900 KB) [16] are reported but further higher marker density with average spacing of less than 10 KB has not been reported so far.

Our present work aims at development of such first microsatellite marker database based on whole genome based STR mining which is very user-friendly and freely accessible. Also, the feature of user defined primer designing has great advantage in terms of precise selection from each chromosome, from defined location, size of amplicons for ease of rapid genotyping in simple and low cost agarose gel.

## Construction and content

### Database processing pipeline

The chromosome wise tomato whole genome data available in public domain [17] was downloaded in FASTA format. All the 12 available chromosomes of the genome were chopped into manageable range using PERL script to be put into MIcroSAtellite identification (MISA) tool [18]. The information on STR numbers, motifs, repeat number, length and size of the repeat, repeat type, GC content, start and end position of the repeat and STR sequence were compiled. Around 1.4 million STRs were generated from tomato genome. Scripts in PERL were written to arrange the output of MISA in proper format in order to create the data file to be further imported to MYSQL database.

Option to find STRs from tomato genome specifically based on chromosome location, type of motif, repeat motif and repeat kind are available. The advance option for search is also available for STRs in desired range of GC content, number of base pairs and copy number. Further, selected STRs can be used in wet lab by generating primers with the integrated Primer3 standalone tool [19]. This obviates the need of manual primer designing using tool/ server.

#### Database architecture

**Figure 1:**
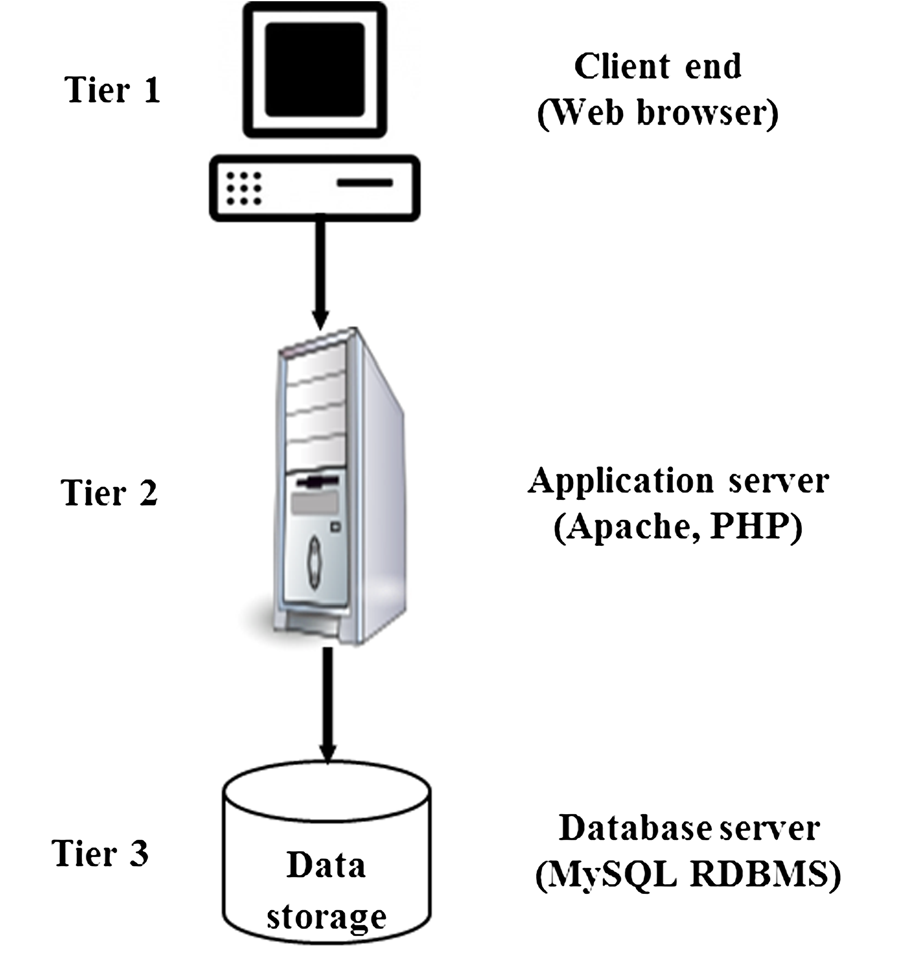
**Three-tier architecture of *TomSatDB.***

Tomato MicroSatellite Database (*TomSatDB)* catalogues all the available information of 1.4 million microsatellite repeats of tomato genome taken under study. It is an online relational database with “three-tier architecture” (Figure 1) with a client tier, middle tier and database tier. In first tier, the total *in silico* STRs mined using MISA is stored in MySQL database. The middle tier flexibility provision according to the user need has been given. Also, the primer3 standalone code has been integrated to compute primers on user request. The third tier of architecture i.e. client end gives the list of multiple primers along with melting temperature, GC content, start position and product size of selected STRs. The use of open-source server-side scripting language i.e. PHP (Hypertext Pre Processor) has been employed to develop this user friendly interface of *TomSatDB.*

*TomSatDB* has seven tabs (Home, About, Database, Analysis, Tutorial, Links and Team) where general information of the developed tomato microsatellite database, tomato, microsatellite markers, analysis of the tomato genome have been described. Tutorial contains the guidelines for users and terminologies used in the database contents. *TomSatDB* houses other useful links related to solanaceae family.

#### Accessing database

The *TomSatDB* is very flexible and easy to handle where the user may query for microsatellites over 12 chromosomes, either one or more chromosomes being selected at a time from tomato genome. The searches may further be customized based on various microsatellite characteristics like motif type (mono, di, tri, tetra, penta, hexa), repeat motif and repeat kind (simple and composite).

**Figure 2:**
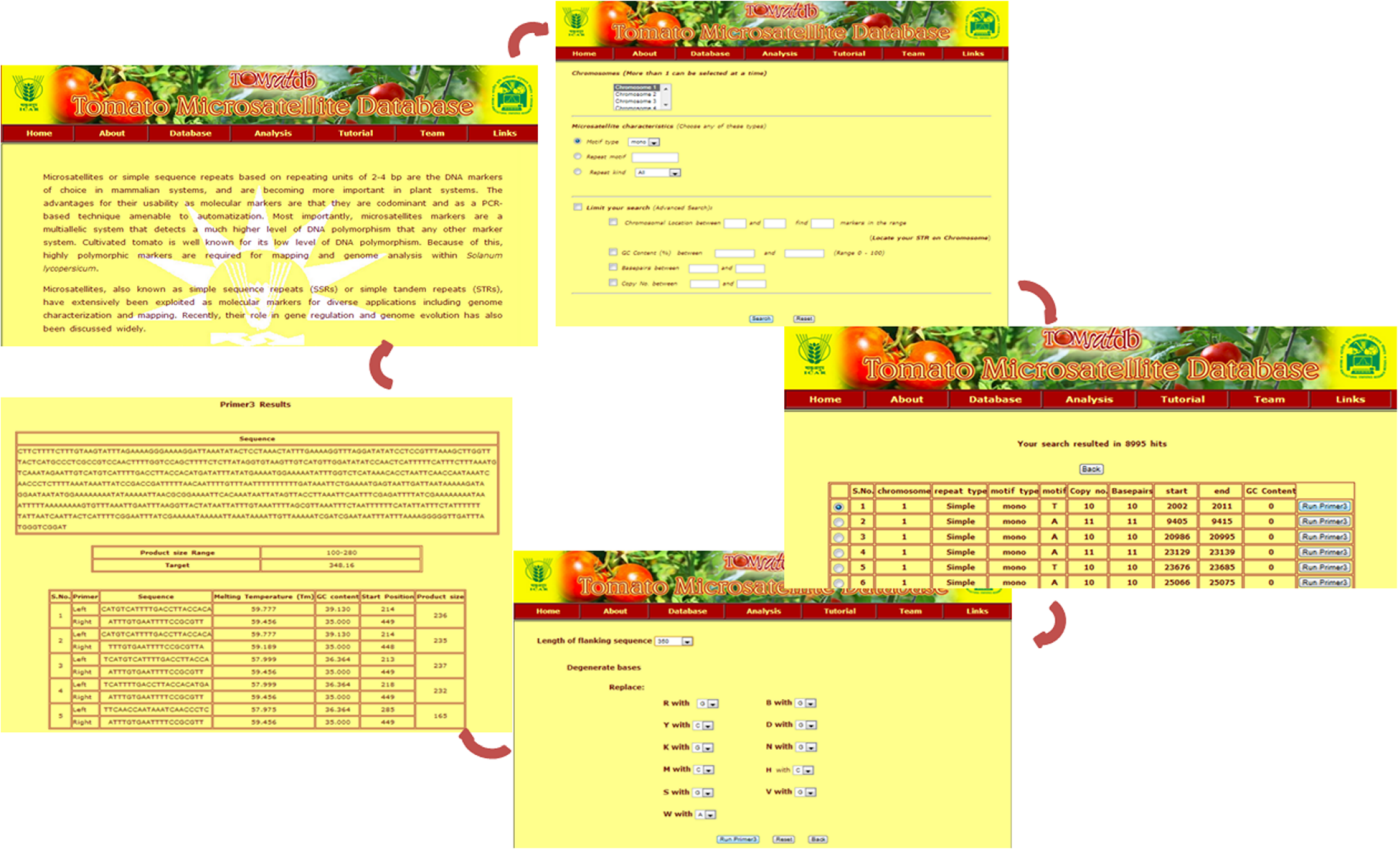
**Flow of search at *TomSatDB.***

The user may go for advance search by specifying the location of STRs on chromosome, number of markers in the given range, markers within given range of GC content, number of basepairs and copy numbers. The STRs preferably at equal interval essentially help in identification of QTL and fine mapping of economically important genes based on LOD scores. Figure 2 shows the flow of database search.

Primer3 tool [19] has been integrated in *TomSatDB* to generate primers of selected STRs. The STRs may be selected with the help of radio button for generation of primers. Provision to design primer for selected STR locus is provided with a template of approximately 1000 base pairs by selecting upto 500 base pairs of both flanking regions. The provided flexibilities would enable researchers to select markers at known location over the desired chromosomes.

Further, each individual STR of a targeted region over chromosome may be used to narrow down location of gene of interest or linked QTL. The users are given flexibility to replace degenerate bases with any of the alternative bases (A,T,G,C) in *TomSatDB*.

### Analysis of tomato genome and relative abundance

The whole genome was analyzed for getting an overview of the tomato genome. It was observed that 87% and 13% of the STR markers were of simple and compound type, respectively. The “mono” repeat type (50.17%) was found to be dominant followed by “di” (26.69%) type (Table 1).The number of “hexa” repeat type (153) was found to be minimum (0.10%). A considerable variation in microsatellite motif length classes in genomes from species to species has been reported [20]. Abundance of di-nucleotide repeats in eukaryotic genome are reported [21,22] but in our data “mono” repeats are most abundant due to inherent limitation of the NGS technology having more mono nucleotide stretches as sequencing error [23]. The longer the chromosome, proportionately higher is the total repeat content as expected in ubiquitously distributed STR markers [24]. The length of STRs between 9–16 was found to be most occurring (58.08%) followed by 5–8 and >16 as 22.06% and 6.63% respectively of total STR markers. Chromosome 1 is having the highest number of markers while chromosome 6 exhibits minimum number of STR markers. Chromosome 8 shows highest density (177.8 markers/MBp) of markers and chromosome 2 reports minimum density of markers (128.4 markers/MBp), while the relative density of the tomato whole genome is 154.3 markers/MBp, showing that these markers are ubiquitously distributed with homogeneity in terms of distance, which is inherent attribute of microsatellite to be used as marker of choice.

**Table 1:** Chromosome wise distribution of STRs

**Chromosomes**	**Simple**						**Compound**	**Total**
	**Mono**	**Di**	**Tri**	**Tetra**	**Penta**	**Hexa**		
Chromosome 1	8995	4632	1476	194	35	19	2311	17662
Chromosome 2	6401	2746	867	95	15	6	1363	11493
Chromosome 3	6637	3370	1107	150	18	21	1572	12875
Chromosome 4	6332	3268	1076	115	24	13	1499	12327
Chromosome 5	5714	3300	1028	128	17	11	1632	11830
Chromosome 6	5105	2652	861	102	8	9	1219	9956
Chromosome 7	5801	3097	1045	137	22	11	1580	11693
Chromosome 8	5974	3052	966	136	19	11	1556	11714
Chromosome 9	5886	3275	1098	118	20	17	1613	12027
Chromosome 10	5369	3041	910	140	15	12	1417	10904
Chromosome 11	5005	2640	916	91	16	3	1360	10031
Chromosome 12	5365	3034	995	126	20	18	1687	11245
Chromosome 0	972	1018	231	33	3	2	586	2845
**Total**	**73556**	**39125**	**12576**	**1565**	**232**	**153**	**19395**	**146602**

### STR validation

Finding of these *in silico* mined STR markers needs extensive wet lab validation across all important tomato varieties of the world. An attempt was made for preliminary small *in silico* validation with available markers of SGN database [14] using PERL script (Table 2). We found extremely low matching of primers (11.71%). The potential reasons for this magnitude in *in silico* validation could be varietal difference, ESTs derived STRs and potential of null alleles in tomato genome (*Heinz* variety taken under study). Though some of the primers from crops other than tomato from solanaceae family showed positive validation up to 7.19% which is obviously expected in different species due to null alleles and genomic changes during speciation in heterologous mode use of STR [25].

**Table 2:** STR validation result of primers from Solgenomics

**http://STR Markers from http://solgenomics.net/**		
	**Tomato**	**Others**
Total no. of primers reported	1383	1668
No. of positive primers	162 (11.71%)	120 (7.19%)

## Utility and discussion

Limited attempt of STR development was reported for example, 12^th^ chromosome has just 37 markers by earlier *in vivo* method, but *in silico* method has extracted 11245 from very same chromosome giving much more marker density, which is highly desirable [12]. Though the genome size range of solanaceae is varying from 0.950 GB (tomato) to 2.70 GB (capsicum) but this family has fixed 12 chromosomes. Although the gene repertoire and gene order of solanaceae species are well conserved, the cause of the genome-size difference is not known [26].

STR plays important role in mapping, trait improvement, variety development, variety identification and product traceability. Traditionally, characterization of varieties is based on phenotypic observation but it is very difficult to distinguish varieties with very similar morphological characteristics and identification of the cultivars accurately is essential for maintaining cultivar integrity and Plant Breeders’ Rights.

Limited studies have been reported in variety identification of tomato using STR DNA markers. In one study, out of 20 STR markers, only 11 were able to discriminate 47 varieties [27] and in another study, 12 markers could differentiate 34 varieties [28]. Studies based on 6000 SNP markers over 93 varieties have demonstrated that SNP based variety differentiation is also possible [29]. However, in such SNP based studies, the genotyping data of "Moneymaker" and "Moneyberg" varieties were completely identical leading to no differentiation at all. So, more STR markers from tomato genome are warranted to address varietal differentiation and product tractability in the food chain. Also, DNA fingerprinting is an appropriate tool to track and trace the tomato supply chain, ensuring not only authenticity and integrity of the products but also the absence of any possible genetic contamination by other species or unwanted components [30-32].

Such use of STR in plant variety identification is well reported in other crops like barley varieties [33], *S. tuberosum ssp. tuberosum*[34], sugarcane [35], capsicum [36], eggplant [37] and identification of Basmati rice from that of non-Basmati rice [13]. Also, the microsatellite STR markers are the method of first choice to complement the DUS (Distinctness, Uniformity and Stability) testing procedure [38,39].

Our STR database can be a useful tool in MAS programme of tomato improvement. Such use of STR in crop improvement is already reported in sorghum [40], tagging stem rust resistance gene Sr35 in wheat [41], *Fusarium* head blight resistance in wheat [42], leaf rust resistance gene Lr35 in wheat [43] and mapping of resistance gene effective against Karnal bunt pathogen of wheat [44]. Wheat improvement programs to enhance leaf rust resistance using STR markers has been attempted [45]. STR markers are also used for introgression programs for trait improvement, for example Soltol QTLs in rice. The location of the Saltol QTL on chromosome 1 and identification of additional QTLs associated with salt tolerance is well identified [46].

The relative density of the tomato whole genome reported in the study is 154 markers per MBp. This is almost in range with Arabidopsis (157 MBp), the other crops reported are with higher number of markers like cucumber (367 MBp), rice (370–490 MBp), popular (485 MBp), grape (487 MBp), sorghum (818 MBp), soybean (1115 MBp), maize (2365 MBp), wheat (1000 MBp) and pigeon pea (833 MBp). Though a general negative correlation between genome size and STR density in plants has been reported [47] but we found distance between markers are not proportion to size of genome thus small genome size has enough marker density for mapping purpose.

*TomSatDB* is of great use to tomato breeders in molecular breeding. The customization of this tool for search based on chromosome may be used by breeders for mapping of gene by markers. It is likely to be accessed by biologists engaged in research with diverse objectives in the crop primarily to develop molecular markers and also to understand the functional significance of microsatellites in regulating gene expression and genome evolution. The comprehensive options to search for simple and compound microsatellites repeats in the genic regions allow users to explore new avenues of investigations on these repeats. The primer designing for PCR amplification of desired motifs will facilitate studies on mutability, microsatellite abundance etc. Association of microsatellites with a particular disease or phenotype may also be explored. Microsatellite data can also be used to investigate various anomalies using candidate gene approach. This microsatellite database will serve as an important application for extracting information in order to design experiments in new directions elucidating novel roles and functions of microsatellites. The STR markers (>1.4 m) reported here is not only relevant for tomato germplasm management using MAS against 200 biotic and abiotic stress but also to other crops. This database is expected to be of immense use across globe by respective statutory authorities for variety identification and varietal dispute resolution supplementing DUS test and product traceability.

## Conclusions

A total of 146602 STR markers are reported for the first time using whole genome in the database. Though we have reported small attempt of *in silico* validation in our studies but extensive wet lab validation of these markers is warranted. These markers are expected to pave the way of germplasm management over abiotic and biotic stress as well as improvement through molecular breeding, leading to increased tomato productivity in various parts of the world. The marker reported in our database are ready to use for mapping as well as also for variety identification and product traceability, paving the pathway of best use of genomics and computational tool in endeavor of tomato improvement and variety management at global level.

## Availability and requirement

*TomSatDB,* the tomato microsatellite marker database is freely accessible for research purposes for non-profit and academic organizations at http://cabindb.iasri.res.in/tomsatdb.

## Competing interests

The authors declare that they have no competing interests.

## Authors’ contributions

DK conceived this study. MAI, S and VA created the work-flow, database, web-tool and performed data analyses. MAI, S, NV, DK and AR drafted the manuscript. All authors read and approved the manuscript.
